# TiRobot‐Assisted Percutaneous Cannulated Screw Fixation for Elderly Patients with Fragility Fractures of the Pelvis: A Retrospective Study

**DOI:** 10.1111/os.14011

**Published:** 2024-02-21

**Authors:** Baorui Xing, Xiaoyu Shen, Lijie Ma, Xiangbei Qi

**Affiliations:** ^1^ Department of Orthopedic Surgery The Third Hospital of Hebei Medical University Shijiazhuang China; ^2^ Department of Orthopaedic Surgery Hebei Cangzhou Hospital of Integrated Traditional Chinese and Western Medicine Cangzhou China

**Keywords:** elderly patients, fragility fracture of the pelvis, percutaneous cannulated screw fixation, TiRobot‐assisted surgery, treatment

## Abstract

**Objective:**

The incidence of fragility fractures of the pelvis (FFPs) is increasing in the elderly population, and FFPs that require fixation are a challenge for orthopedic surgeons. The insertion of implants is not risk free due to the complex anatomical and osteoporotic bones and requires a steep learning curve. This study aimed to investigate the clinical efficacy of TiRobot‐assisted percutaneous cannulated screw fixation in the treatment of elderly FFP patients.

**Method:**

The clinical data of 46 elderly FFP patients who had been treated with percutaneous cannulated screw fixation from May 2020 to September 2022 were retrospectively analyzed. Twenty‐four patients were treated with percutaneous cannulated screw fixation assisted by the TiRobot (TiRobot‐assisted group) and 22 patients were treated with conventional freehand surgery (freehand group). Postoperative outcomes, including Matta value, excellent and good rate (EGR) of fracture reduction, and accuracy of screw placement (ASP), were compared. Changes in the Visual analog scale (VAS) pain score and the Majeed score were recorded and compared between groups before and after surgery and during the 24‐week follow‐up. Repeated‐measures analysis of variance (ANOVA) and effect sizes were used as analysis methods.

**Results:**

A total of 90 screws were implanted, 51 screws in the TiRobot‐assisted group and 39 screws in the freehand group. The operation time of the two groups was 34.1 ± 2.67 min versus 64.5 ± 4.19 min (*p* < 0.001). There were no screw‐related complications or revision surgeries in any group. The Matta value of the TiRobot‐assisted group was 5.13 ± 3.52, which was significantly lower than that of the freehand group (9.00 ± 3.68, *p* < 0.001), while the EGR was 91.67% versus 72.73%, with statistical significance (*p* < 0.001). The ASP was 100% in the TiRobot‐assisted group, better than that in the freehand group, where it was 85.7% (*p* = 0.043). At each timepoint in the early postoperative period, the VAS score of the TiRobot‐assisted group was significantly lower than that of the freehand group and was close to consistent by the last follow‐up; the Majeed score of the former was significantly higher than that of the latter at each timepoint of follow‐up, with statistical significance (*p* < 0.001).

**Conclusion:**

TiRobot‐assisted percutaneous cannulated screw fixation of elderly FFP patients is advantageous over conventional freehand surgery, with less invasion, more accurate screw placement, better fracture reduction, early pain relief, and rapid recovery, suggesting that Freehand method to stabilize FFP in the elderly population.

## Graphical abstract

First, the “human tracer” (Track) was placed in the iliac region and C‐arm anterior‐lateral X‐rays were taken. A three‐dimensional (3D) image of the pelvic region was captured and transmitted to the main screen of the Tianji robot. Second, the “simulation” mode was selected and the robot arm was run to the target position. A surgical incision of 0.5–1.0 cm was made on the skin of the body along the position shown by the robotic arm. The muscle tissue was bluntly separated until it reached the bone surface, and the guide needle sleeve was inserted until the tip of the sleeve touched the bone surface. Third, the automatic execution mode of the TiRobot 2.0 system was adopted. Automatic simulation was based on the current position of the robot arm. Guide pins and hollow screws were placed in sequence along the sleeve direction according to the pre‐planned length and direction. A 3D image was then taken.

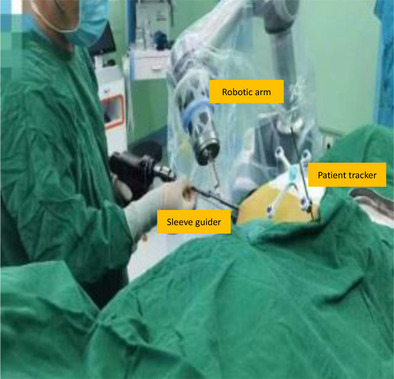

## Introduction

Fragility fractures of the pelvis (FFPs), also known as osteoporotic pelvic fractures, have become one of the most common entities affecting the health of the older population worldwide.^1^, ^2^ With the advent of the aging population, the incidence of osteoporosis in the elderly has increased, and the incidence of FFP has increased yearly.^3^ There are reports that the number of cases will increase 2.4 times by 2030.^4^ Unlike traumatic pelvic fractures in general, FFP often results from a low‐energy violent injury and is characterized by intense pain, loss of mobility, and diminution of independence. Treatment options include surgical and conservative treatment. The aim is to relieve pain as soon as possible, restore mobility, and prevent and control complications. Given the high complication rate and mortality rate within 1 year after injury following conservative treatment, most scholars prefer surgical treatment for these patients. A retrospective study of the clinical data of 128 patients with FFP showed that active surgical treatment can reduce the complications related to bed rest after fracture and significantly reduce mortality rates.^5^ However, the anatomical structure of the pelvis is complex, the superior rami of the pubis is irregular, and the curve and inclination of the bony channel vary greatly in the anteroposterior plane and the coronal plane. The traditional hands‐free nail placement is difficult, the learning period is long, the radiation exposure to the surgeon and the patient is considerable, and the risk of screw perforation through bone is high.^6^, ^7^ The incidence of plant penetration through bone and invasion of the joint during percutaneous screw fixation is 0.9% to 7%.^8^, ^9^ One study reported that among 50 FFP patients with an average age of 79 years and 1‐year follow‐up who were treated with CT‐guided percutaneous sacroiliac screw, 41 cases recovered well. In 23 patients who underwent bilateral sacroiliac joint surgery, only two screws were loosened.^10^ Noser et al. treated 60 patients with FFP with percutaneous sacroiliac screws. During a 62‐month follow‐up, the mean Majeed score of the surviving patients was 65 (85.5% of the maximum).^11^ Over the years, in treatment this disease, the surgical mode has changed from the traditional open surgery with high risk and many complications to minimally invasive surgery, and manual screw implantation and internal fixation has been widely used in clinical practice. In recent years, robot‐assisted surgery has been applied to a variety of orthopedic procedures, including joint replacement, spine surgery, bone tumor surgery, arthroscopic surgery, and fracture fixation,^12^, ^13^, ^14^, ^15^, ^16^, ^17^ and has achieved accelerated postoperative recovery.

Currently, there are several internal fixation methods to treat FFP in the elderly. First, traditional open reduction internal fixation has the advantages of good reduction and solid fixation, but there are problems, such as greater surgical trauma and higher risk of incision infection. Second, infix (minimally invasive anterior pelvic ring internal fixator) is a minimally invasive surgery for pelvic fractures, but it also has issues, such as the long learning curve, lateral femoral cutaneous nerve injury, and femoral nerve paralysis.^18^, ^19^ Third, lumbosacral or sacroiliac screw is a safe and effective fixation method for posterior pelvic ring injury.^20^ However, the position of the fixed screw needs to be repeatedly verified by fluoroscopy. There is a risk of damage to blood vessels and nerves, which is a challenge for surgeons. Finally, percutaneous channel screw treatment for pelvic fracture is a central intramedullary fixation approach with obvious shear resistance advantages, showing good biomechanics and good clinical characteristics of rapid postoperative rehabilitation. However, the screw placement operation technology and accuracy requirements are very high. Conventional manual procedures require repeated fluoroscopy and are associated with radiation exposure to both the doctor and the patient, a high risk of perforation of the bone cortex or acetabulum, and an increased likelihood of structural damage to nerves, blood vessels, and other components. Despite the increasing incidence of FFP in the elderly population, evidence on the most appropriate treatment for FFP remains limited. The frailty of these elderly patients with FFPs requires specific, less invasive treatment algorithms. As such, to address these challenges, robot‐assisted surgery has been applied in the field of traumatic orthopedics.

At present, most hospitals still use conventional freehand screw placement under fluoroscopy for osteoporotic pelvic internal fixation. With the introduction of the TiRobot system in our hospital, we carried out TiRobot‐assisted percutaneous cannulated screw fixation for elderly FFP patients and achieved preliminary clinical results. The purpose of this study was to assess (i) the clinical efficacy of TiRobot‐assisted percutaneous cannulated screw fixation in the treatment of elderly patients with FFP and (ii) the superiority, limitations, and precautions in the application of this surgical procedure.

## Methods

### Inclusion and Exclusion Criteria

The inclusion criteria included: (i) patients who were at least 65 years old with osteoporotic pelvic fractures; (ii) patients who had a bone mineral density test T value ranging from −3.5 SD to −2.5 SD (−3.5 SD ≤ T value ≤ −2.5 SD) and a fresh fracture of FFP classification III–IV; (iii) patients who had the bone mineral density test T value ranging from −3.5 SD to −2.5 SD (−3.5 SD ≤ T value≤ −2.5 SD) and FFP type I–II patients who received conservative treatment aggravated pain within the first week or inability to stay in bed for long periods of time^21^; (iv) patients who had undergone TiRobot‐assisted percutaneous cannulated screw fixation or conventional freehand surgery; (v) follow‐up time ≥6 months; (vi) complete clinical data was available. Exclusion criteria were patients: (i) with an open or obsolete fracture; (ii) with pathological fractures (e.g., bone metastasis of cancer, primary bone tumor, and metabolic bone disease); (iii) who could not tolerate the operation because of comorbidities, such as serious heart, lung, liver, kidney, and other internal diseases; (iv) who could not cooperate with treatment (including rehabilitation) due to mental illness; and (v) who could not undergo closed reduction because of multiple injuries or severe osteoporotic pelvic fractures.

### General Information of Patients

This retrospective study reviewed a case series from May 2020 to September 2022. The study protocol was approved by our Institutional Review Board (CZX2023‐KY‐011). A total of 46 patients were admitted in this study. Preoperative X‐rays CT scans of all patients were used to evaluate the management protocol for pelvic fractures.

Twenty‐four patients, including 8 men and 16 women, ranging in age from 65 to 87 years with a mean age of 73.21 years, were treated with TiRobot‐assisted percutaneous cannulated screw fixation (TiRobot‐assisted group). Twenty‐two patients who had undergone conventional surgery with manual positioning during the same time period were selected as the control group (the freehand group). Among them, there were seven men and 15 females, ranging in age from 66 to 88 years, with an average age of 74.14 years. According to FFP classification,^22^ FFP Type I refers to the anterior injury only, including Ia and Ib. FFP Type II refers to the non‐displaced posterior injury, including IIa, IIb, and IIc. FFP Type III refers to the displaced unilateral posterior injury, including IIIa, IIIb, and IIIc. FFP Type IV refers to a displaced bilateral posterior injury, including IVa, IVb, and IVc. According to the FFP classification, the number of eligible patients in both groups was four versus five for type I, 12 versus 10 for type II, three versus four for type III, and five versus three for type IV. The patient characteristics are shown in Table 1.

**TABLE 1:** Baseline demographic and clinical characteristics of the included participants

Characteristics	TiRobot‐assisted group (*n* = 24)	Freehand group (*n* = 22)	*t*/*χ* ^2^	*p*‐value
Age, mean (SD), years	73.21 (6.29)	74.14 (7.32)	*t* = 0.459	0.648
Gender			*χ* ^2^ = 0.012	0.913
Female	16	15		
Male	8	7		
BMD‐T value, mean (SD)	−3.12 (0.27)	−3.09 (0.26)	*t* = −0.381	0.705
FFP classification			*χ* ^2^ = 0.012	0.913
I	4	5		
II	12	10		
III	3	4		
IV	5	3		
VAS score, mean (SD)	5.46 (1.20)	5.27 (1.35)	*t* = −0.507	0.615
Majeed score, mean (SD)	37.21 (3.73)	36. 05 (2.87)	*t* = −1.191	0.240

Abbreviations: BMD‐T, bone mineral density test value; FFP, fragility fracture of the pelvis; VAS score, Visual analogue scale score; Majeed score, Majeed pelvic outcome score (a functional scoring system specifically developed for pelvic injuries, with an overall value of 100 points).

### Surgical Equipment and Procedure (TiRobot‐Assisted)

Twenty‐four patients in the TiRobot‐assisted surgery group received percutaneous cannulated screw fixation treatment. The operation was performed with the assistance of TiRobot, the second generation of the orthopedic surgical robot of Beijing TINAVI Medical Technology (TiRobot ForcePro Superior, China). To check whether the robot is fully equipped before operation, routine preoperative preparation is required for the workstation, optical tracking system, robotic arm, C‐arm X‐ray machine, and other equipment. After the equipment is connected and powered on, it is necessary to check and debug the equipment to ensure that it is operating properly. The surgeon then logs into the system, records medical records, and selects surgical tools.

### Surgical Procedure

**FIGURE 1:**
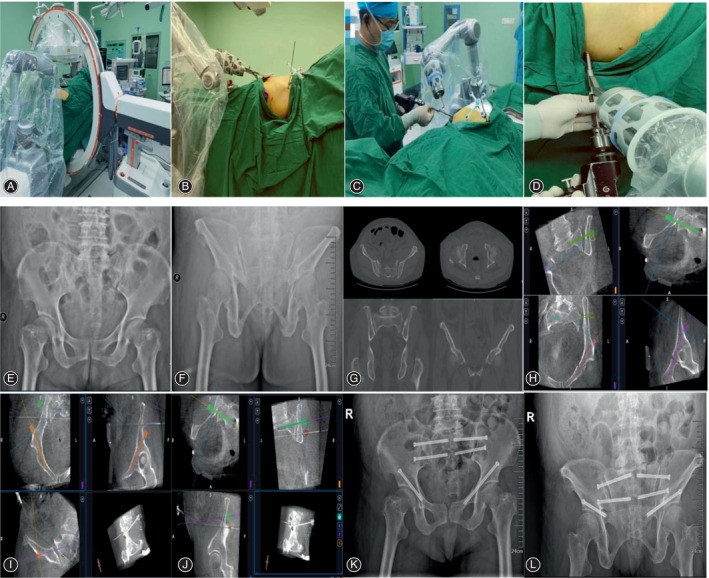
A 73‐year‐old woman with a fragility fracture of the pelvis (FFP IVa type). The surgical procedure of TiRobot‐assisted channel screw fixation. (A,B) C‐arm was used to capture intraoperative three‐dimensional (3D) images. (C,D) The surgeon placed the screws according to the 3D guide of the Tianji robotic arm, and the screw diameter was 6.5 mm to assist indirectly the fracture reduction. (E,F) Preoperative anteroposterior (AP) and outlet radiographs of the pelvis showed bilateral sacroiliac joint separation and bilateral acetabular anterior column fracture. (G) Horizontal and coronal CT of the pelvis: bilateral sacroiliac joint separation, left sacral fracture, bilateral sacroiliac joint “open‐book” injury, and bilateral acetabular anterior column fracture. (H–J) On the host interface of the “Tianji” orthopedic robot, screw placement for the sacroiliac joint was planned on both sides (S1/S2), and screw placement for the anteroposteric column was planned on both sides. Screw positions and lengths could be detected on 3D multi‐plane and 3D fluoroscopic images. (K,L) Postoperatively, anteroposterior (AP) and outlet pelvic radiographs showed good fracture reduction and internal fixation. Bilateral sacroiliac joint screws and bilateral anterograde anterior column screws were in good positions and did not invade the peri‐channel cortical bone.

**FIGURE 2:**
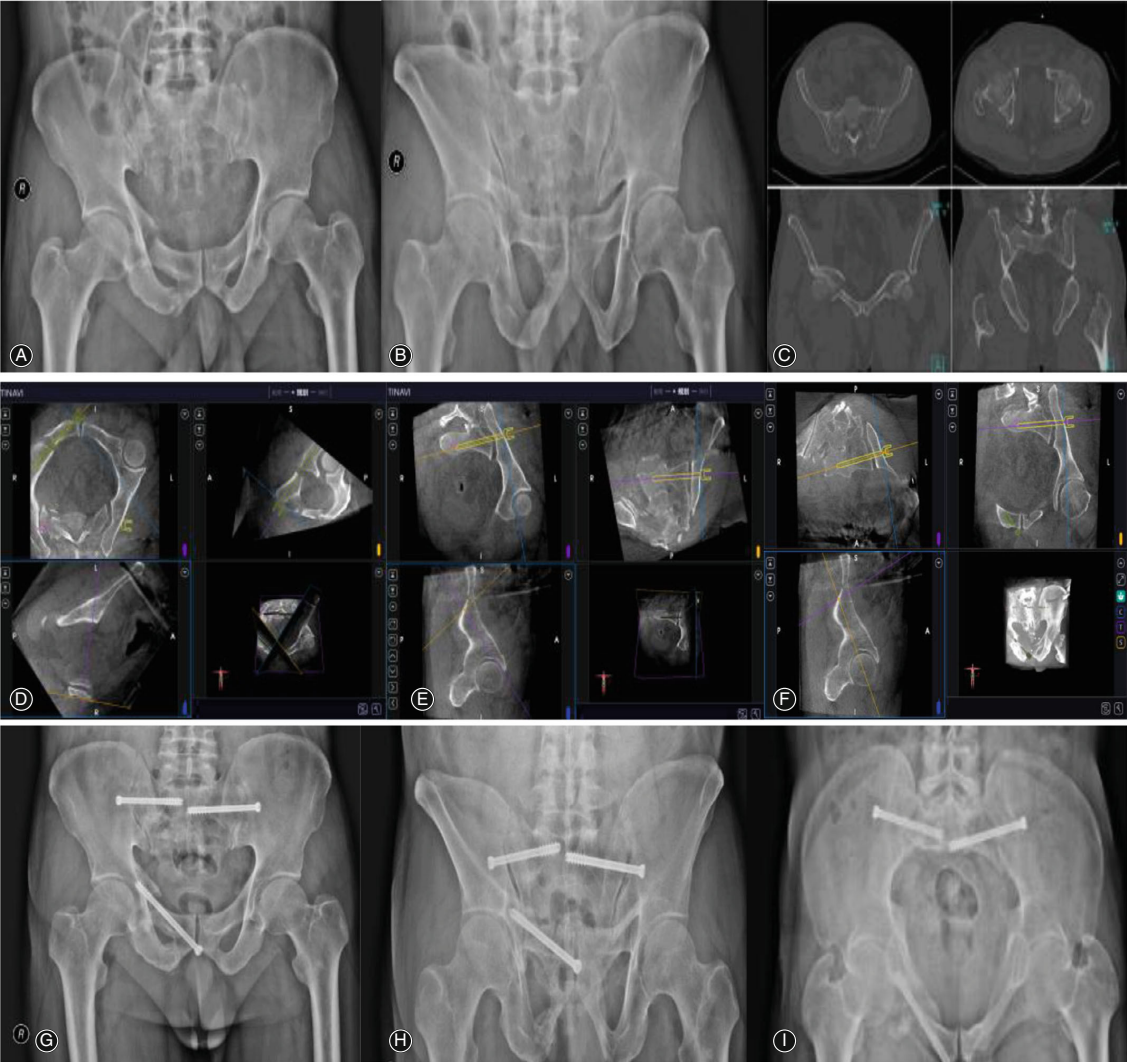
A 67‐year‐old man with a fragility fracture of the pelvis (FFP IVc type). Preoperative, and intraoperative planning and postoperative images of TiRobot‐assisted channel screw fixation. (A,B) Preoperative anteroposterior (AP) and outlet radiographs of the pelvis show the bilateral sacroiliac joint separation and the right acetabular anterior column fracture. (C) Horizontal and coronal CT of the pelvis showed a “close‐book” injury of the right sacroiliac joint, a “crescent” fracture of the right posterior iliac bone, a compression fracture of the Dennis II region of the right sacrum, a partially overlapping and displaced fracture of the right superior pubic branch (anterior acetabular column) and subpubic branch, an “open‐book” manifestation of the left sacroiliac joint, and a non‐displaced fracture of the left superior pubic branch (anterior acetabular column). (D–F) Intraoperative planning images. (G–I) Postoperatively, anteroposterior (AP), outlet, and inlet radiographs of the pelvis showed good reduction and internal fixation. Bilateral sacroiliac joint screws and right anterograde anterior column screws were in good position.

After administering the general anesthesia, the patient was placed in a supine position and underwent routine disinfection and covering. For type III and IV pelvic fractures with obvious displacement, bone traction reduction, indirect obstruction reduction with different diameters of guide needle channel and screw channel planned by Tianji Robot, and limited incision minimally invasive reduction of the pelvis were used for closed reduction.^1^ For three‐dimensional (3D) image acquisition, the robot, C‐arm machine, and optical tracking camera were placed in an appropriate position. The “Human tracer (Track)” was placed in the iliac region without interfering with the operation, and anteroposterior and lateral C‐arm X‐rays were taken to confirm that the calibration point of the scale and the bone structure of the proposed nail were included in the collected images. The 3D image of the pelvic region was captured and transmitted to the main screen of the Tianji robot.^2^ For path planning and cannulated screw placement, the appropriate entry point was selected as well as the direction to complete the planning of the target screw. “Simulation” mode was selected and the robot arm was run to the target position. A surgical incision of 0.5 – 1 cm was made on the skin of the body along the position shown by the robotic arm. The muscle tissue was bluntly separated until it reached the bone surface, and the guide needle sleeve was inserted until the tip of the sleeve touched the bone surface. The automatic execution mode of the TiRobot 2.0 system was adopted. The software execution interface is available after the end function is enabled (enabled by default). After the foot pedal was connected, automatic simulation was performed based on the current position of the robot arm. Guide needle and cannulated screws were placed in turn along the direction of the sleeve according to the pre‐planned length and direction. A 3D image was then taken. The incision was closed and covered with a sterile dressing. All operations were performed by three pelvic trauma orthopedic surgeons, all of whom were trained by professional intelligent robots. Figure 1 shows a female patient (FFP IVa type) to illustrate the surgical procedure. Preoperative, postoperative, and intraoperative images of another male patient (FFP IVc type) are shown in Figure 2.

### Surgical Operation (Conventional)

The patient was placed in a supine position and lateral C‐arm X‐ray fluoroscopy of the pelvis was performed. Lateral images of the first sacral vertebra and Iliac Cortical Dencity (ICD) projection line were located on the affected side. A 1‐cm incision was made, and the guide needle was implanted into the sacral vertebra channel under fluoroscopy. Then, the C‐arm X‐ray fluoroscopy was used to repeatedly view the entrance and exit positions of the first sacral vertebra of the pelvis; intermittent dynamic fluoroscopy was performed to monitor whether the guide needle was drilled into the sacroiliac joint and the sacral vertebral body to the midline. The position of the guide needle was satisfactory in the lateral, entrance, and exit positions of the pelvis under fluoroscopy. After measuring the length, a cannulated screw was inserted and fixed. Re‐fluoroscopy was performed, and the incision was sutured after it was deemed satisfactory.

### Outcome Parameters

Matta value was used to evaluate the quality of fracture reduction based on radiographic measurements.^23^ The post‐treatment radiograph was evaluated based on Matta criteria: A (excellent, ≤4 mm fracture gap or step‐off deformity), B (good, 5 – 10 mm gap or step off), C (fair, 11 – 20 mm gap or step off), and D (poor, >20 mm gap or step off). The excellent and good rate (EGR) of fracture reduction refers to “A + B”/“A + B + C + D” × 100%.

Gras criteria^24^ were used to evaluate the accuracy of screw placement (ASP). The position of the screw implant was assessed using three categories: class I (excellent, secure placement, completely in the cancellous bone), class II (fair, secure placement, but contacting the cortical bone), and class III (poor, malplacement, penetrating the cortical bone). ASP refers to the “number of screws of class I + number of screws of class II”/“total number of implanted screws” × 100%.

The visual analog scale (VAS) pain score was a common method to evaluate the intensity of subjective pain and the degree of psychological sensation. In clinical assessment, a score of 1–3 indicates mild pain; a score of 4–6 indicates moderate pain; and a score of 7–10 indicates severe pain.^25^ The lower the score, the milder the symptoms.

The Majeed score was used to assess the postoperative recovery of pelvis function. The score system includes seven aspects: pain, working, sitting, standing, walking distance, independent gait, and sexual function. The maximum score is 100 points (the best possible outcome). Total scores of <70, 70–80, 80–90, and 90–100 indicate poor, fair, good, and excellent outcomes, respectively.^26^

The fluoroscopy frequency (total number of intraoperative fluoroscopy images taken; i.e., the fluoroscopy frequency of the surgeons and the patient), fluoroscopy dose (duration of each fluoroscopy; i.e., the fluoroscopy exposure time of the surgeons and the patient), operation time (total time to complete surgical procedures), and blood loss (the amount of blood lost during the operation) were the secondary outcomes.

### Postoperative Treatment and Follow‐Up

The postoperative treatment was similar between the two groups. These patients were given analgesic treatment, anticoagulant drugs, lower extremity pressure pump, ankle pump training, and other measures to prevent lower extremity deep vein thrombosis. Salmon calcitonin nasal spray can be used for postoperative anti‐osteoporosis treatment. Prophylactic anti‐infection therapy was given within 24 h after surgery. The postoperative pelvic radiographs and CT scans were performed for postoperative evaluation of the reduction and screw placement. Starting 24 h after surgery, patients were assisted with mild hip flexion activities two to three times a day and guided to exercise lower limb strength. By 2 weeks after surgery, the patient was allowed to actively bend the hip and knees in bed. At 4 weeks after surgery, the patient could perform partial weight‐bearing exercises with the help of a walker. By 12 weeks after surgery, when the fracture line was significantly blurred, the patient was allowed to perform complete weight‐bearing exercises. Matta value and Gras criteria were used to evaluate the quality of the fracture reduction and the accuracy of screw placement. During the 24‐week follow‐up period, the VAS score was used to continuously record and evaluate pain changes in patients, while the Majeed score was used to evaluate the process and outcome of postoperative recovery of pelvis function. The patients were surveyed on the first, seventh, 28th, 84th, and 168th postoperative days within 24 h. The fracture healing and associated adverse events were recorded, including fracture re‐displacement, channel screw loosening, wound infection, hypostatic pneumonia, lower extremity deep vein thrombosis, malunion, and nonunion.

### Statistical Analysis

Stata 17.0 version (Stata Corp LP) was used to analyze the study data. The measurement data were tested by Kolmogorov–Smirnov normal analysis. Normally distributed data were conveyed as mean (SD); the *t*‐test was applied for evaluation between the two cohorts. The non‐parametric Mann–Whitney *U*‐test was applied for non‐normally distributed data. The data among all groups were compared by χ^2^‐test. If the theoretical frequency was too small, Fisher's exact probability method was used. The changes from baseline in mean VAS score and Majeed score were analyzed using repeated‐measures ANOVA. Effect sizes are described as Cohen's *d*. All statistical analyses were two sided, and *p*‐values less than 0.05 were considered indicative of statistically significant difference.

## Results

Between May 2020 and September 2022, we enrolled a total of 61 patients, 15 of whom were excluded from the study. Forty‐six eligible patients were treated with screw implantation; 51 screws were used in TiRobot‐assisted group and 39 screws were used in the freehand group. All patients were followed up for 6 months and were re‐examined every 4–6 weeks. In both groups, there were no consequences from cannulated screw loosening, fracture re‐displacement, vascular injury, wound infection, malunion, or nonunion.

### Intraoperative Outcome Indicators

The number and duration of intraoperative fluoroscopy images taken in the TiRobot‐assisted group were 6.12 ± 1.19 T and 5.91 ± 1.24 S, respectively, significantly less than those in the freehand group (18.31 ± 2.10 T and 14.04 ± 2.10 S) (both *p* < 0.001). The operative time of the TiRobot‐assisted group was 34.1 ± 2.67 M, which was significantly shorter than that of the freehand group (64.5 ± 4.19 M) (*p* < 0.001). The amount of surgical bleeding in the TiRobot‐assisted group was 17.04 ± 3.14 mL, which was significantly lower than that in the freehand group (36.22 ± 3.25 mL) (*p* < 0.001). Details are shown in Table 2.

**TABLE 2:** Comparison of intraoperative outcomes between the two groups

Indexes	TiRobot‐assisted group (*n* = 24, mean ± SD)	Freehand group (*n* = 22, mean ± SD)	*t*‐value	*p‐*value
Fluoroscopy frequency (times)	6.12 ± 1.19	18.31 ± 2.10	24.470	<0.001
Fluoroscopy duration (seconds)	5.91 ± 1.24	14.04 ± 2.10	16.097	<0.001
Operation time (min)	34.1 ± 2.67	64.5 ± 4.19	29.630	<0.001
Blood loss (mL)	17.04 ± 3.14	36.22 ± 3.25	20.352	<0.001

### Fracture Reduction

**FIGURE 3:**
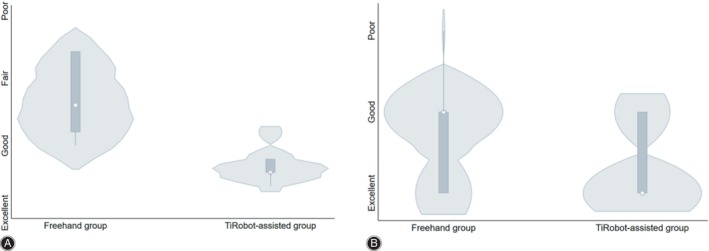
Violin plot of reduction quality and screw placement accuracy postoperation. (A) Matta criteria. (B) Gras criteria.

The fracture reduction effect was evaluated by Matta criteria. The EGR (FFP type III and IV) were 87.50% in the TiRobot‐assisted group (two excellent cases, five good cases, and one fair case) and 28.57% in the freehand group (one excellent case, one good case, four fair cases and one poor case). The difference between the two groups was statistically significant (*p* < 0.001), as shown in Table 3 and Figure 3(A). Based on the Matta value, the effect of fracture reduction in the TiRobot‐assisted group was significantly better than that in the freehand group (*p* < 0.001), as seen in Table 3.

**TABLE 3:** Evaluation of fracture reduction and screw placement in the two groups

Index	TiRobot‐assisted group number of cases (screws)^a^	Freehand group number of cases (screws)^a^	*χ* ^2^‐value	*p‐*value
Matta criteria A/B/C/D (EGR)	2/5/1/0^b^ (87.50%)	1/1/4/1^b^ (28.57%)	–	0.041^c^
Gras criteria I/II/III (ASP)	16 (41)/8 (10)/0 (0) (100%)	6 (14)/13 (20)/3 (5) (87.18%)	4.695	0.043

Abbreviations: Matta criteria (A/B/C/D), the postoperative radiograph (excellent/good/fair/poor); EGR, the excellent and good rate of fracture reduction; Gras criteria (I/II/III), CT scans of screw placement (excellent/good/poor); ASP, the accuracy of screw placement.

^a^ (screws) Number of implanted screws.

^b^ Type III and IV cases.

^c^ Fisher's exact.

### Accuracy of Screw Placement

According to the Gras criteria of the affected pelvic fractures, the 51 screws implanted in the TiRobot‐assisted group were classified as follows: class I,^41^ class II,^10^ and class III (0); the ASP was 100%. Thirty‐nine screws implanted in the freehand group, 22 screws were defined as class I, class II,^12^ and class III.^5^ The ASP was 87.18% (*χ* ^2^ = 4.695, *p* = 0.043). There was a statistically significant difference between the two groups, as shown in Table 3 and Figure 3(B). This means that the TiRobot‐assisted screw implantation had greater accuracy.

### Visual Analog Scale

**FIGURE 4:**
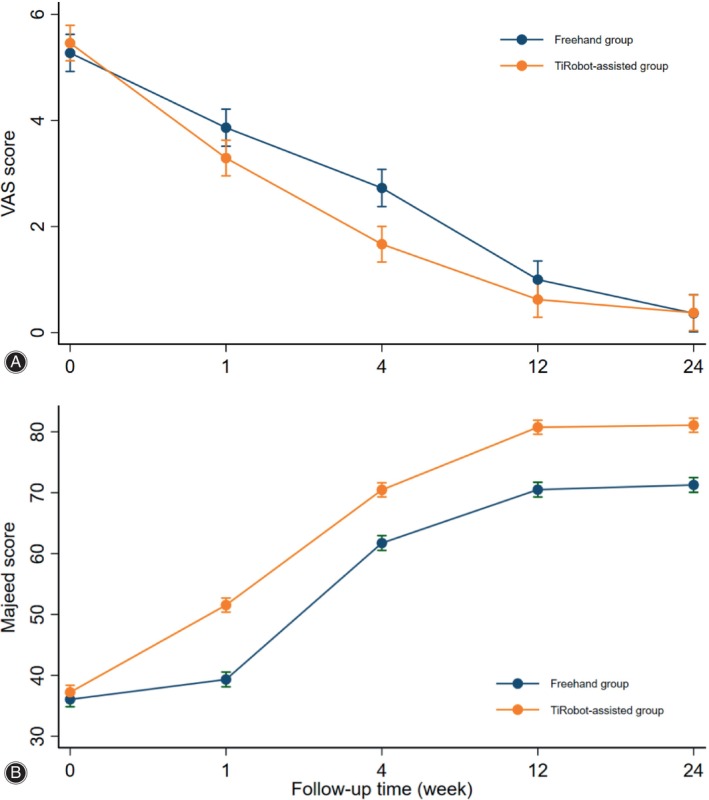
Therapeutic Effects of TiRobot‐assisted channel screw fixation. (A) Visual analog scale (VAS) scores of the two groups at different time points during the follow‐up period. (B) Majeed scores of the two groups at different time points during the follow‐up period.

The VAS scores at 1 week, 4 weeks, 3 months, and 6 months after treatment were lower than the preoperative scores of the same group. At 1 week after treatment, the VAS score of the TiRobot‐assisted group was 3.29 ± 0.81 points, which was lower than that of the freehand group (3.86 ± 0.94 points) (*p* = 0.033). The VAS score at 4 weeks after treatment was 1.67 ± 0.76 points in the TiRobot‐assisted group, which was lower than the 2.73 ± 1.03 points in the freehand group (*p* < 0.001). There was no significant difference in VAS scores between the two groups after 12 weeks of follow‐up. For both groups, *p* > 0.05; see Table 4 for details. The changes in VAS scores between the two groups are shown in Figure 4(A).

### Pelvic Function Recovery

There was no significant difference in Majeed scores between the two groups preoperation (Table 1). The Majeed scores at 1 week, 4 weeks, 12 weeks, and 24 weeks after treatment were higher than those preoperation in the same group (all *p* < 0.05). Notably, the Majeed score of the TiRobot‐assisted group was higher than that of the freehand group at all time points during the follow‐up period. There was a statistically significant difference between the two groups (all *p* < 0.001), as seen in Table 4. The changes in Majeed scores between the two groups is shown in Figure 4(B).

## Discussion

Our research revealed that TiRobot‐assisted percutaneous cannulated screw fixation for elderly FFP patients had the advantages of being precise, accurate, and minimally invasive and having a short operation time, low radiation damage, and rapid functional recovery. It was also significantly superior to the freehand surgery group in terms of fracture reduction and early pain relief. There were no screw‐related complications or revision surgeries in any group. The results of this retrospective study suggest that TiRobot‐assisted surgery is a better method to stabilize FFP in the elderly population.

### Efficacy and Safety of TiRobot‐Assisted Surgery

Wu *et al*.^27^ were the first to use the Tianjin orthopedic robot system to carry out robot‐assisted percutaneous minimally invasive pelvic fixation. Compared with the traditional manual operation, it can make the screw implantation more accurate and can greatly reduce the intraoperative X‐ray radiation exposure, shorten the operation time and reduce the amount of blood loss. Liu *et al*.^28^ applied the Tianji orthopedic robot in the minimally invasive treatment of pelvic posterior ring and anterior ring fractures, demonstrating that robot‐assisted patients could significantly reduce intraoperative bleeding and move early after surgery. Most patients had achieved good healing at 3–6 month postoperative follow‐up. Long *et al*.^13^ performed sacroiliac joint screw fixation with the assistance of the Tianji robot. They conducted a comparative study with the traditional freehand operation, showing that the robot group was superior to the traditional operation group in terms of intraoperative fluoroscopy times and total duration, operation time, incision size, and anesthesia time. To date, however, there is no standard surgical procedure for FFP, and appropriate studies are few or pending.

In recent years, with the development of the concept of minimally invasive surgery and related techniques, minimally invasive percutaneous sacroiliac screw fixation has been widely used in the treatment of pelvic fractures. In particular, the computer‐guided surgery robot and the assistant application of 3D imaging technology have greatly enhanced the accuracy of screw placement and improved the ability of orthopedic doctors to place screws, it plays an important role in improving the effect and safety of minimally invasive surgery for pelvic fractures. In our study, the TiRobot system successfully assisted percutaneous cannulated screw fixation of FFP with intuitive surgical path planning. Indeed, screw implantation fixation is a safe surgical procedure for stabilizing FFP. During the 24 weeks of follow‐up, there were no complications such as screw loosening, secondary screw displacement wound infection, vascular or nerve injury, malunion, or nonunion in our study. Similar to other studies,^29^, ^30^, ^31^, ^32^, ^33^, ^34^ we found that compared with the conventional freehand operation, TiRobot‐assisted surgery has obvious advantages in fracture reduction, accuracy, stability, early pain relief, and rapid recovery.

The Matta score of fracture reduction quality after treatment in the TiRobot‐assisted group was 87.50%, which was much higher than that in the freehand group (28.57%). The EGR of reduction was mainly due to the fact that we used the TiRobot system to plan indirect barrier reduction of the broken end of the fracture with different diameters of the guide needle channel and the screw channel, achieving the integration of the minimally invasive concept and indirect reduction, which was the first attempt to treat pelvic fractures with cannulated screws. In fact, there is no consensus on whether robot‐assisted surgery can effectively adjust fracture reduction in FFP treatment. A study by Han *et al*. (2022) came to a different conclusion, arguing that robot‐assisted surgery does not affect reduction rates.^35^ Due to the relative lack of relevant research data, there is little reference for our results. Nevertheless, we believe that the use of TiRobot‐assisted surgery is beneficial for fracture reduction in FFP patients.

According to the Gras criteria of the affected pelvic fractures, the ASP in the TiRobot‐assisted group was 100% (24/24), much higher than 87.18% in the freehand group. In the treatment of elderly FFP with the assistance of the second‐generation TiRobot (Tianji 2.0 system automatic execution mode), we carried out a micro‐adjustment of the whole process of intraoperative needle implantation, thus achieving accurate and reliable screw placement. In the actual operation process, the procedure reduced the risk of decreasing the holding force of the channel screw after repeated freehand adjustment and also avoided the slight swing of the guide needle of the X‐ray navigation sleeve of the O‐arm with the fluctuation of the patient's breathing and pulse. Precise positioning, accurate placement, and stable fixation are the main goals of internal fixation of pelvic fractures using the medical screws. Based on our results, compared with manual surgery, TiRobot‐assisted surgery is more conducive to achieving this goal. Supporting this notion is the fact that Gras classification measurements show a 100% screw implantation accuracy rate.

In this study, preoperative mean pain levels were similar between the two groups, with no significant difference. From the first week of postoperative follow‐up, VAS scores showed statistical difference between the two groups, and the pain level in the TiRobot‐assisted group was significantly lower than that in the freehand group. It was not until 24 weeks after surgery that the pain scores of the two groups were nearly consistent. The VAS score in the TiRobot‐assisted group decreased from 4.5 before surgery to 2.1, which is in line with the results other studies, where the VAS score varies from 0.36 to 3.4.^36^, ^37^, ^38^, ^39^, ^40^, ^41^ Follow‐up VAS scores indicated that TiRobotic‐assisted surgery was more effective in relieving early pain in FFP patients. It is true that TiRobotic‐assisted surgery achieves the goal of early pain relief for FFP patients. Further, it reduces the incidence of cardiovascular and cerebrovascular events caused by pain stimulation and promotes the functional recovery of lower limbs.

As an important parameter value for evaluating the recovery of pelvic function after surgery, we found that Majeed scores in the TiRobot‐assisted group were higher than those in the freehand group at each follow‐up time point (1 week, 1 month, 3 months, and 6 months). At the last follow‐up, the Majeed scores of the two groups were 81.09 (SD 2.78) versus 71.27 (SD 4.44). The results of this study suggest that robotic surgery for the treatment of osteoporotic pelvic fractures in the elderly contributes to early recovery of pelvic function. In other words, it might support the rapid and comprehensive recovery of FFP patients. A recent study in China showed that robot‐assisted surgery has obvious advantages in terms of accuracy, stability, and reduction of intraoperative radiation exposure, but the benefits are still inconclusive in terms of functional recovery.^12^ We believe that our findings can be used as an update and supplement to the above conclusions.

Additionally, we found that the TiRobot‐assisted group is significantly better than the freehand group in terms of the fluoroscopy frequency of the surgeons and the patient, the fluoroscopy exposure time of the surgeons and the patient, operation time, and blood lost, indicating its advantages in reducing trauma and radiation damage. Several studies have reported results consistent with our findings.^2^, ^12^, ^35^

### Advantages of TiRobot‐Assisted Surgery for Fragility Fractures of the Pelvis

Percutaneous sacroiliac screw fixation is particularly beneficial in the treatment of fragility pelvic fractures in elderly patients with osteoporosis, as well as in polytraumatized patients who benefit from prompt operative management.^42^ Elderly patients with FFP have weakened physiological function and often have pre‐existing underlying diseases. The incidence of surgical complications under conventional open reduction treatment can be as high as 43.9%.^43^ Minimally invasive percutaneous channel screw technology is increasingly used in pelvic fractures.^44^ With the decrease of bone mineral content, FFP displacement can cause bone channel narrowing. It has been reported that even 5 mm displacement in sacral fractures can reduce the S1 screw channel by 36%.^45^ Failure to locate too narrow bony channels can result in channel screws penetrating the bone cortex and causing peripheral nerve and vascular damage. To improve the safety and accuracy of the operation, various navigation systems have been used in clinic to assist the fixation of pelvic fractures and dislocations.^46^ The early experiences and learning curve of orthopedic robot‐assisted spinal fracture surgery have been reported in the published literature, but there is little research on the application of robot‐assisted pelvic fracture surgery.^47^ Wong *et al*. used 3D navigation technology to perform percutaneous internal fixation of pubic ramus screw in 17 patients with FFP.^39^ Elderly FFP is an indication for TiRobot‐assisted percutaneous cannulated screw fixation. In fact, the final outcomes were that in the treatment of elderly FFP, TiRobot‐assisted surgery was superior to conventional manual surgery in fracture reduction, screw implantation fixation accuracy, early postoperative pain relief, pelvic function recovery, and other aspects. Its advantages are summarized as follows. The first is precise positioning and accurate placement of the implants, such as cannulated screws. Specifically, the robot provides precise spatial positioning and stable path navigation for screw placement, and through the movement of the robotic arm, the guide screw is accurately, safely, and stably placed at the anatomical site. The second advantage is programmed surgery. The process only needs to capture 3D images, complete path planning, and drilling positioning. By following the procedure, the guide needle can be inserted precisely in the planned position. The use of 3D intraoperative imaging allowed us to assess complex anatomies, perform surgery on displaced fractures, and obtain real‐time data during and after fracture reduction. That data, in turn, was used for robotic insertion of implants.^48^ Indeed, the use of intraoperative 3D imaging has been shown to improve cannulated screw placement. The third advantage is its correction function. Once the actual path of the guide pin is found to deviate from the planned path, the fine‐tuning function of the software can be used to adjust the angle of the robot arm, thus effectively ensuring the safety of the operation. The fourth advantage is the reduction of radiation damage. Compared with manual screw insertion, the robotic procedure significantly reduces the cumulative intraoperative radiation dosage (Table 2).

**TABLE 4:** The treatment effects for the TiRobot‐assisted group and freehand group

Index	TiRobot‐assisted group (*n* = 24)	Freehand group (*n* = 22)	Difference (95% CI)	*p* value	Effect size^a^
Matta's value (SD)					
Posttreatment	3.50 (1.19)^b^	8.43 (2.94)^c^	4.93 (2.17 to 7.68)	0.003	2.26
VAS score (SD)					
1 week posttreatment	3.29 (0.81)	3.86 (0.94)	0.57 (0.05 to 1.09)	0.033	0.66
4 weeks posttreatment	1.67 (0.76)	2.73 (1.03)	1.06 (0.52 to 1.61)	<0.001	1.18
12 weeks posttreatment	0.63 (0.65)	1.00 (0.76)	0.38 (0.05 to 0.79)	0.079	0.53
24 weeks posttreatment	0.38 (0.49)	0.36 (0.49)	−0.01 (−0.30 to 0.28)	0.938	−0.02
Majeed score (SD)					
1 week posttreatment	51.54 (2.26)	39.32 (1.87)	−12.22 (−13.45 to −10.99)	<0.001	−5.87
4 weeks posttreatment	70.46 (2.19)	61.73 (3.06)	−8.73 (−10.33 to −7.13)	<0.001	−3.31
12 weeks posttreatment	80.75 (2.71)	70.50 (4.22)	−10.25 (−12.39 to −8.11)	<0.001	−2.92
24 weeks posttreatment	81.09 (2.78)	71.27 (4.44)	−9.81 (−12.05 to −7.57)	<0.001	−2.67

Abbreviations: Matta's criteria, imaging evaluation of reduction quality after internal fixation of pelvic fractures (indicating the size of the fracture gap or step off); SD, standard deviation; CI, confidence interval; VAS score, visual analogue scale score; Majeed score, Majeed pelvic outcome score (a functional scoring system specifically developed for pelvic injuries, with an overall value of 100 points).

^a^ Effect sizes are listed as Cohen's *d*.

^b^ (*n* = 8).

^c^ (*n* = 7).

### Precautions and Improvements in the TiRobot‐Assisted Surgical Procedure

The second generation of the Tianji orthopedic robot‐assisted treatment for elderly FFP patients has the advantages of precision and being minimally invasive and intelligent, paving the way for the treatment of elderly FFP, but there will be some problems in the specific implementation process. We need to solve these problems to, for instance, take advantage of orthopedic robots and avoid and reduce repeated fluoroscopy, poor reduction, and screw implantation errors.

First, we used a C‐arm to capture 3D images. Intraoperative images of the sacroiliac, anterior column, and bilateral pelvic structures might not be obtained at the same time, so the patient might not be located in the center of the carbon fiber fluoroscopy bed. Reasonable arrangements should be made according to the patient's injured site, screw placement planning, C‐arm placement, and so forth to obtain the bone structure of the effective surgical area and to arrange screw planning and placement of the anterior and posterior pelvic rings. Of course, if the hospital has advanced equipment, the O‐arm navigation system can present high‐definition 3D images for the surgeon, clearly showing the degree of fracture reduction, whether the articular surface is flat, and whether the screw position and length are accurate so that the surgery is more minimally invasive, accurate, and intelligent. It improves surgical safety and reduces doctor–patient radiation exposure, laying the foundation for obtaining a satisfactory curative effect.

Second, for the Tianji orthopedic robot, keeping the human tracer (Track) relatively stable with the target bone structure of the patient is the premise of the accuracy of the cannulated screw, and ensuring the correct real‐time correlation of the human tracer, fracture target bone structure, optical tracking camera, and robotic arm tracking during surgical operation are key to the robot completing the screw placement according to the plan.

Third, osteoporotic pelvic fractures are characterized by thinner than normal cortical bone and more irregular pelvic structure. Therefore, the risk of deviating from the set entry point, penetrating the contralateral cortex, and damaging blood vessels and nerves is higher than that of normal bone tissue. The intraoperative procedure requires blunt separation to the bone surface, not to the smooth bone cortex. The principle of “fast drilling and slow entry” should be observed to avoid excessive resistance into the needle. The principle of “let nature take its course” should be followed through the cancellous bone screw channel. The length of the insertion guide needle: the distance between the tip of the handheld electric drill and the end of the guide sleeve was planned according to the image to limit the depth of the guide needle to avoid the risk of penetrating the contralateral cortex of the bone and damaging blood vessels, nerves, and other organs.

### Study Limitations and Prospects for Clinical Application

First, the retrospective nature of this study is a limitation. Second, the sample size for this study was small. Third, the follow‐up period in this study was relatively short. In addition, the study did not examine biomechanics. Future multicenter, large sample prospective studies are necessary, and guidelines for robot‐assisted minimally invasive pelvic fracture treatment need to be developed.

Currently, robot‐assisted surgery in the clinical application of trauma orthopedics in China is developing rapidly. Further, robot‐assisted trauma orthopedic surgery, such as screw implantation and fixation of pelvic fractures, has become a research hotspot worldwide.^49^ Compared with traditional surgical methods, it has obvious advantages in accuracy, stability, low radiation damage, and rapid functional recovery. Therefore, we have reason to believe that trauma surgery robots have broad application prospects in the future, especially for pelvic fracture surgery.

## Conclusion

Minimally invasive screw fixation is an acceptable treatment for FFP in elderly patients. The TiRobot‐assisted surgery provides precise spatial positioning and stable path navigation for screw placement in elderly patients with FFP, overcoming the shortcomings of the conventional manual surgical methods, such as unstable manual operation, and more radiation damage. It has the advantages of good fracture reduction, higher accuracy, less invasion, early pain relief, and quick postoperative recovery and is a manual operation for FFP in elderly patients. Based on our findings, the TiRobot‐assisted surgery has great clinical application value for the treatment of elderly FFP patients.

## Author Contributions

Baorui Xing, Lijie Ma, and Xiangbei Qi carried out the study design and writing of the manuscript. Baorui Xing, Xiangbei Qi, Lijie Ma, and Xiaoyu Shen confirmed the completeness and validity of the data. Baorui Xing and Xiaoyu Shen participated in the data collection and analysis. Baorui Xing, Xiangbei Qi, and Lijie Ma conceived of the study, participated in its study design, and revised the manuscript. All authors reviewed and approved the final manuscript.

## Conflict of Interest Statement

The authors declare no potential conflicts of interest with respect to the research, authorship, and publication of this article.

## Ethics Statement

This study was performed in line with the Declaration of Helsinki. Approval was granted by the Ethic Committee of Hebei Cangzhou Hospital of Integrated Traditional Chinese and Western Medicine, China (CZX‐2023‐KY‐011).

## Data Availability

The datasets used and/or analyzed during the current study are available from the corresponding author on reasonable request.
